# Validation of Cell-Based Assay for Quantification of Sesamol Uptake and Its Application for Measuring Target Exposure

**DOI:** 10.3390/molecules24193522

**Published:** 2019-09-28

**Authors:** Tarapong Srisongkram, Natthida Weerapreeyakul

**Affiliations:** 1 Graduate School (in the program of Research and Development in Pharmaceuticals), Faculty of Pharmaceutical Sciences, Khon Kaen University, Khon Kaen 40002, Thailand; tarapong.sri@gmail.com; 2 Division of Pharmaceutical Chemistry, Faculty of Pharmaceutical Sciences, Khon Kaen University, Khon Kaen 40002, Thailand; 3 Human High Performance and Health Promotion (HHP&HP) Research Institute, Khon Kaen University, Khon Kaen 40002, Thailand

**Keywords:** *Sesamum indicum*, Pedaliaceae, sesamol, HPLC, method validation, melanoma, cell-based assay, intracellular concentration

## Abstract

The intracellular drug concentration is needed for determination of target exposure at the site of action regarding its pharmacological action and adverse effects. Sesamol is an antiproliferative molecule from *Sesamum indicum* with promising health benefits. We present a method for measuring the intracellular sesamol content using reverse-phase HPLC with a UV diode array in melanoma cells. Sesamol was completely resolved by isocratic elution (4.152 ± 0.008 min) with methanol/water (70%, *v*/*v*) through a 30 °C, 5-µm C-18 column and detection at 297 nm. The present assay offers high sensitivity, fast elution, and an accurate and linear nominal concentration range of 10–1000 ng/mL (*R*^2^ = 0.9972). The % accuracy of the sesamol quality control sample was −3.36% to 1.50% (bias) with a 0.84% to 5.28% relative standard deviation (RSD), representing high repeatability and high reproducibility. The % recovery was 94.80% to 99.29%, which determined that there was no loss of sesamol content during the sample preparation. The validated method was applied to monitor intracellular sesamol concentration after treatment from 5 min to 24 h. The remaining intracellular sesamol content was correlated with its antiproliferative effect (*R*^2^ = 0.9483). In conclusion, this assay demonstrated low manipulation, quick elution, and high sensitivity, precision, accuracy, and recovery, and it was successfully applied to the quantification of sesamol in target cells.

## 1. Introduction

Many compounds in pharmaceutical or food product development interact with their targets along with being metabolized inside cells [1]. Ascertaining the respective concentrations of intracellular unbound bioactive compounds is needed to determine bioactivity at the site of action, target exposure, and adverse events [2]. The extracellular level of some molecules does not, however, proxy for respective pharmacological effects, as protein transporters can increase or decrease the level of intracellular compounds [3]. In contrast, intracellular unbound compound concentrations are notoriously difficult (a) to measure in vivo, as only extracellular unbound concentrations (i.e., in plasma) are considered vis-à-vis their pharmacological effects; and (b) to investigate in terms of the target exposure level.

**Figure 1:**
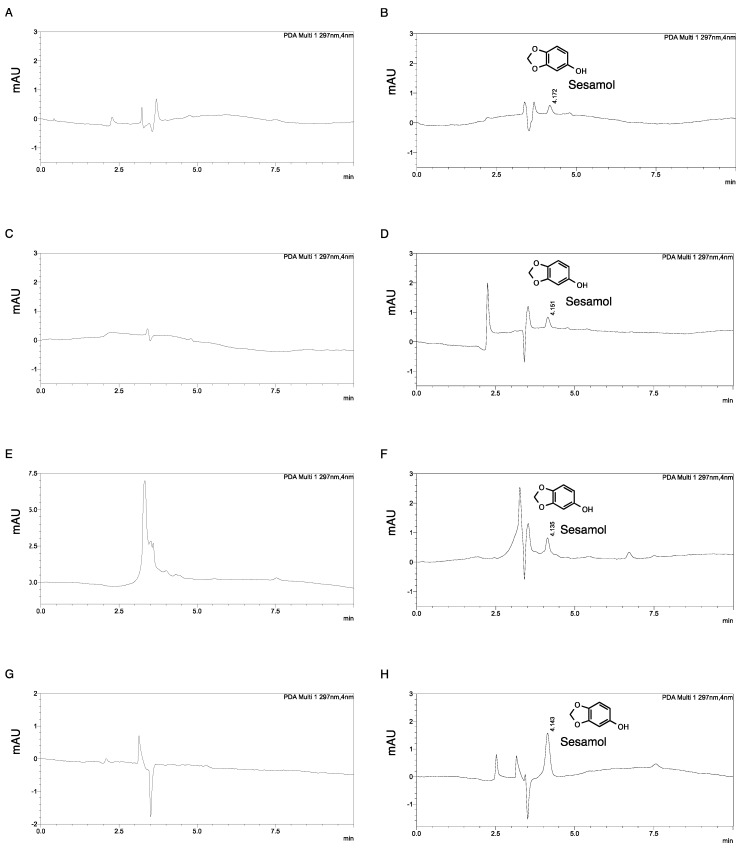
Chromatogram of sesamol in various solvents: (**A**) acetonitrile, (**B**) 20 ng/mL sesamol in acetonitrile, (**C**) 0.1 M sodium hydroxide, (**D**) 20 ng/mL sesamol in 0.1 M sodium hydroxide, (**E**) 100 mM hydrochloric acid, (**F**) 20 ng/mL sesamol in 100 mM of hydrochloric acid, (**G**) methanol, and (**H**) 20 ng/mL sesamol in methanol.

Sesamol (3,4-methylenedioxyphenol) (Figure 1) is found in small amounts in the seed coat and oil of roasted *Sesamum indicum* [4]. Sesamol is a natural phenolic antioxidant with promising health benefits [5]. The antioxidant value of sesamol is greater than the other bioactive constituents of sesame (i.e., sesamin and sesamolin) [6]. The bioactivity of sesamol is in in vivo anti-skin cancer proliferation [7], anti-hepatocellular carcinoma proliferation [8], anti-atherosclerosis [9], and in vitro anti-melanogenesis [10,11]. Sesamol induces mitochondria disruption, arrests cells in the S phase, inhibits autophagy [8], and interacts with DNA [12], resulting in an induction of apoptosis of cancer cells. Sesamol has a low molecular weight (138.29), low lipophilicity (log*P* of 1.29), and high solubility in water (38.8 mg/mL water), with limited oral [13] and skin absorption [14]. On the basis of the physicochemical properties of sesamol and the criteria for drug permeability [15], sesamol may be predicted to have low permeability through the cell membrane. Hence, the intracellular content of sesamol should be determined to predict the precise biological effect of sesamol in its target cells.

Numerous methods have been developed to estimate intracellular unbound compound concentrations [3,16]; however, they tend to be complicated and/or costly. Generally, methods to quantify intracellular compound concentrations include (a) liquid scintillation counting, (b) high-performance liquid chromatography (HPLC), or (c) flow cytometry. Liquid scintillation counting can quantify a beta particle from a radiolabeled analog inside the cells [2], but it has the limitation of relying on scarce, commercially available, radiolabeled compounds. HPLC is a general experimental instrument available to small labs. HPLC has advantages, including its simple analysis and selective quantitative method. Flow cytometry is an alternative method that quantifies the concentration of intracellular fluorescence compounds using their respective spontaneous fluorescent intensities [17]. However, the number of available fluorescent bioactive compounds is limit [16].

Previously, bioavailability parameters obtained from analyses of sesamol in plasma have been reported from different in vivo models [13,18]. However, the quantitative oral bioavailabilities were significantly different due to the different techniques used for analysis [13,18]. Inconsistencies in sample preparation, protein extraction, and the quantitative method affected the quantitative results. Recently, validated and simultaneous analytical methods of extracting sesamol and other lignans from a *Monascus* aged vinegar [19] and sesamol oil [20] were reported. However, in nonbiological matric conditions, the sample preparation and method of quantification are different. Therefore, a validated detection method in biological metrices is still required.

Thus, we herein present a comparatively easy and reliable method for measuring intracellular sesamol concentrations using reverse-phase HPLC with a UV diode array in a human cutaneous melanoma (SK-MEL-2) cell line. According to previous reports, sesamol suppresses cell proliferation and melanogenesis in melanoma SK-MEL-2 cells [10,21]. Importantly, sesamol has the potential to be used as a whitening agent and benefits melanoma treatment. Therefore, melanoma SK-MEL-2 cells were chosen as target cells in this study. This method provided essential information regarding its application in a cell-based assay.

## 2. Results and Discussion

### 2.1. Optimization of Analytical Method

The aim of the present study was to develop a validated, rapid, simple, and sensitive reverse-phase HPLC method with a UV photodiode array detector for the quantitation of intracellular sesamol concentration in cancer cells. The present method relies on the pretreatment of cell samples. For each cell sample, the culture medium was first removed and washed with ice-cold PBS in order to stop the uptake of the compound. In fact, the low temperature limits the active transport of intracellular drug uptake after the treatment [16]. The washing step was done twice to completely eliminate the leftover contaminant compound in the culture medium and on the cell surface.

The lysis solvent was chosen based on the criteria that it could (i) lyse the cells and also dissolve sesamol, (ii) denature and precipitate proteins or cell debris prior to separation by centrifugation, and (iii) give a clear resolution of sesamol peaks in the HPLC chromatogram. Various solvents (i.e., acetonitrile, 1 M sodium hydroxide, methanol, and 0.01 mM hydrochloric acid) were tested, and each solvent gave different solvent peak characteristics (Figure 1). A clear resolution of the sesamol peak was observed in the methanol extraction (Figure 1G,H). Methanol was, therefore, chosen in the study. It should be noted that a clear supernatant was obtained after centrifugation. There was no more filtration, no supernatant concentrated preparation and no reconstitution before the analysis. Hence, the extraction and isolation steps were not time-consuming. A previous study also reported that using methanol as an eluent gave a higher yield than using other organic solvents did (i.e., acetonitrile and ethanol) [20].

**Figure 2:**
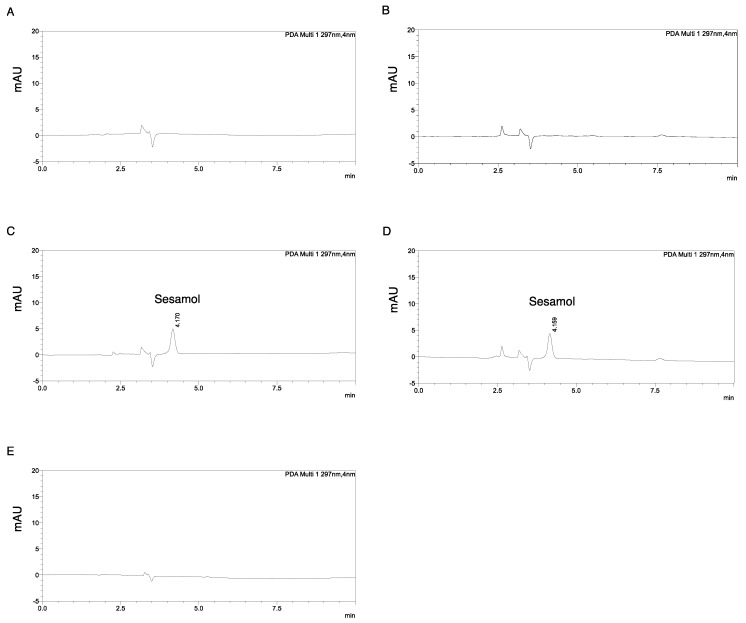
HPLC chromatograms of analytes detected at 297 nm. (**A**) Methanol solvent, (**B**) prepared SK-MEL-2 cell lysate, (**C**) sesamol dissolved in methanol (final concentration of 900 ng/mL), and (**D**) SK-MEL-2 cell lysate spiked with sesamol (final concentration of 900 ng/mL). Additional peaks of sesamol in (**C**) and (**D**) appeared at retention times of 4.152 ± 0.008 min (*n* = 10). (**E**) Carryover effect showed no leftover peak of sesamol after an injection of 900 ng/mL sesamol for 10 min followed by methanol solvent.

The chromatographic elution conditions were performed in a C_18_ base-deactivated reverse-phase silica column. Various mobile phase systems, different flow rates, and column temperatures were optimized. A sharp and symmetric sesamol peak was obtained with the following conditions: 0.8 mL/min and 30 °C for the column temperature. Isocratic 70% methanol was chosen to keep the use of organic solvent at a minimum, and sesamol was rapidly eluted at 4.152 ± 0.008 min (Figure 2). The total analytical run was set at 10 min to ensure that there was no other peak left after sesamol elution. This HPLC condition can be simply set in a basic laboratory.

### 2.2. Validation of Analytical Method

#### 2.2.1. Selectivity and Carryover Effect

Selectivity is the ability of an analytical method to differentiate and quantify an analyte in the presence of other components in a sample [22]. Figure 2 illustrates an analytical HPLC chromatogram of (A) the methanol solvent front (~3.5 min), (B) the SK-MEL-2 cell lysate (~3.5 min), (C) 900 ng/mL of sesamol standard dissolved in methanol, and (D) 900 ng/mL sesamol dissolved in methanol spiked in cell lysate. The sesamol peak (retention time of 4.152 ± 0.008 min) was clearly separated from the solvent peaks. A perfect match to the sesamol peak was observed for each concentration of sesamol standard (peak area: 42,567) and sesamol-spiked cell lysate (peak area: 41,203) (Figure 2C,D). A good separation of the sesamol peak from the endogenous peak of the cell lysate was observed in the chromatogram, indicating good selectivity of the method.

Previous studies have reported the retention times of sesamol from reverse-phase HPLC and LC–MS/MS methods to be 7.441 min [19] and 10.86 min, respectively [23]. Our present analytical method offers a faster elution time of sesamol (less than 5 min). Furthermore, no carryover effect was observed after the analysis of 900 ng/mL of sesamol in cell lysate following the methanol blank sample injection (Figure 2E) and after 10 min of analysis of the methanol injection.

#### 2.2.2. Linearity, LOD, LOQ, and LLOQ

**Figure 3:**
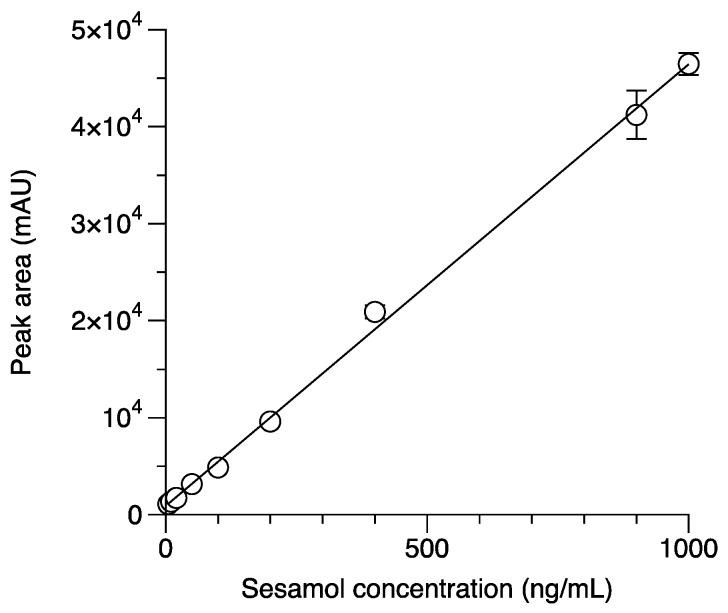
Correlation between the standard sesamol in a range of 10 ng/mL to 1000 ng/mL and the peak area. The average linear regression formula is *Y* = 913.28 (±81.94) + 45.54 (±1.65)*X* with a 0.9972 (±0.0017) coefficient of detection (*R*^2^). The data are from three independent assays.

The linearity of the method was evaluated by fitting a linear equation between the peak areas, and the nominal concentration of the sesamol standard ranged from 10 to 1000 ng/mL. The deviations of actual concentration ranged from −14.14% to 10.44%, and for the lowest concentration, they ranged from −8.78% to −4.41% (Table 1). In all cases, the deviation of the standard curve met the U.S. FDA bioanalytical method validation criteria (within ±15% of the actual concentration of the standard curve) [22], and thus the standard curve was accepted to measure the precisions and accuracies. Linear regression analyses from three separate experiments on the sesamol standard were calculated, which yielded the following formula: *Y* = 913.28 (±81.94) + 45.54 (±1.65)*X*. The coefficient of determination (*R*^2^) of 0.9972 (±0.0017) indicated that the peak area and nominal concentration of the sesamol standard were highly correlated and linear (Figure 3). The lowest dilution of the sesamol standard that gave a reliable detection was at 5 ng/mL. The lowest limit of quantification (LLOQ) was found to be 10 ng/mL, with a % deviation less than or equal to −8.78, while the % residual standard deviation (RSD) values for within-days and between-days were 3.57 and 7.87, respectively (Table 2). The LLOQ can be considered equal to the limit of quantification (LOQ), as previously described [22,24]. The values of the limit of detection (LOD) and the limit of quantification (LOQ) for the quantitation of sesamol by reverse-phase HPLC have been previously reported [18,19]. The LOD and LOQ values reported by Chen et al. (2018) were 20 and 90 ng/mL [19], respectively, and those reported by Geetha et al. (2015) were 10 and 50 ng/mL [18], respectively. The present analytical method had a higher sensitivity for the quantitation of the intracellular sesamol level than did the previous reports because it demonstrated lower LOD and LOQ values.

#### 2.2.3. Recovery

Recovery of the quality control (QC) samples is presented in Table 3. The data are presented as a percentage ratio of the quantitative QC samples and standard solutions. The recoveries from the low, mid-, and high QCs were close to 100% (between 94.80% and 99.29%). The recovery of sesamol in cell lysate was high and close to 100%, indicating no sesamol was lost during the sample preparation process. The high % recovery is a distinct advantage, as it is not necessary to use internal standards [16].

#### 2.2.4. Precision and Accuracy

The precision and accuracy values are summarized in Table 4. The respective within- and between-day precisions of all QC samples—expressed as % RSD—ranged between 0.84% and 4.57% and 3.96% and 5.28%. The precisions were below 6% at all QC levels, which confirmed high repeatability (within a day) and high reproducibility (between days). The respective within- and between-day accuracies—expressed as % bias—ranged between −3.36% and −0.41%, and −2.93% and 1.50%. These values were below 4% at all QC level. Both the precision and accuracy thus met the ±15% requirement for method validation by the FDA recommendations. It is evident that our validated method is reliable. The degree of closeness of the experimental sesamol concentration to the known true sesamol concentration (nominal) under optimized conditions indicates the trueness of the analytical method.

### 2.3. Applying the Method: Monitoring the Intracellular Uptake of Sesamol and Correlating It with Its Antiproliferative Effect in SK-MEL-2 Cells

**Figure 4:**
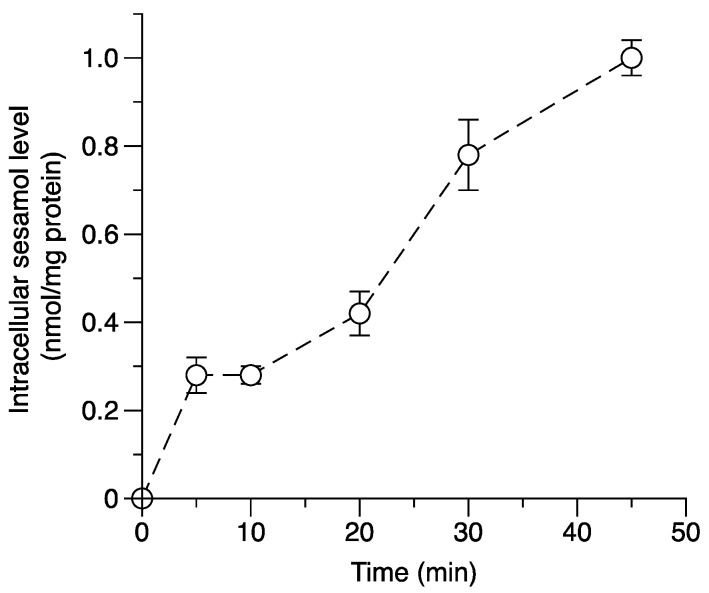
The time course relationship of the intracellular sesamol level (1 mM sesamol in SK-MEL-2 cells). The data were observed from 5 min to 45 min. The data are from three replicates.

**Figure 5:**
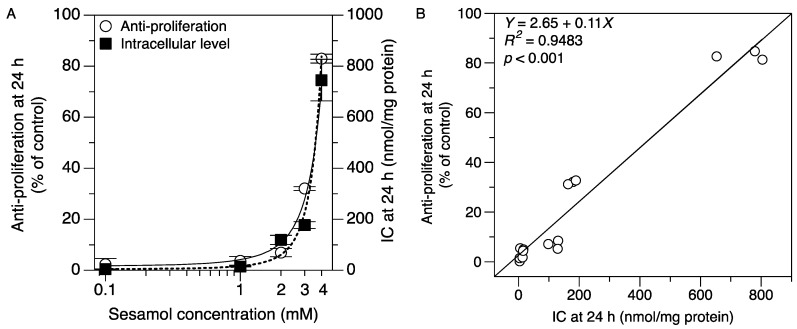
Correlation of intracellular concentration (IC) and antiproliferative effect of sesamol in SK-MEL-2 cells at 24 h. (**A**) The intracellular sesamol concentration (■) and antiproliferative effect (○) were fitted by an exponential model. (**B**) The linear regression analysis between intracellular concentration and the antiproliferation effect at 24 h (*R*^2^ = 0.9483, *p* < 0.001) using a Pearson correlation coefficient. The data were run in triplicate.

The time course of intracellular sesamol uptake was assessed at four different times. The time course of intracellular sesamol concentration is shown in Figure 4. The results show that the intracellular sesamol level dramatically increased from 10 to 45 min in a time-dependent uptake manner. The correlations between intracellular sesamol level and the antiproliferative effect of sesamol were determined to investigate whether the intracellular unbound sesamol concentration could be a potential parameter representing the target exposure of sesamol inside melanoma cells. The antiproliferative effect and intracellular concentration of sesamol in SK-MEL-2 cells were dramatically increased in a concentration-dependent manner (Figure 5A). The target exposure of sesamol inside the SK-MEL-2 cells was strongly correlated with an increasing antiproliferative effect (correlation, *R*^2^ = 0.9483; *p* < 0.001; Figure 5B). The high correlation between the intracellular sesamol and its antiproliferative effect indicated the good prediction of the intracellular concentration parameter. This parameter has demonstrated itself to be a good relative biomarker for determining the antiproliferative effect of a compound [17]. Previously, intracellular drug concentration was introduced as a new predictor for target exposure and target engagement [3,25]. Extracellular concentration (i.e., plasma concentration) does not describe the pharmacodynamic response well if the drug molecules across the cells involve influx and efflux transporters or undergo drug metabolism [3,26]. Our cell-based assay demonstrated that sesamol can engage the target site and remain after the desired period of time (24 h). The remaining amount of sesamol was significantly correlated with its antiproliferation in the SK-MEL-2 cells. This demonstrated free drug exposure at the target site that exceeded pharmacological potency after the desired period, which was key in determining that sesamol achieved adequate target exposure [27].

The described method allowed for the monitoring of the intracellular level of sesamol over time and provided a tool for measuring the compounds inside the cells. Knowing the number of compounds available in the respective target cells is a critical factor for dosage optimization [25]. The prediction of the accurate amount of sesamol at the target will increase accurate dosing or target exposure so as to increase the cellular response, which is a key proxy for successful drug development [27]. In the present study, the validated cell-based assay provided a high recovery percentage with a precise and accurate amount of the quantitative intracellular sesamol level. With its high sensitivity and selectivity, this cell-based assay excludes the confounding factors of invalid analytical methods and the limitations of in vivo pharmacokinetics. Moreover, it offers a low-cost and less invasive validation of the condition compared to optimization using an in vivo study. Therefore, applying an optimized and validated cell-based assay to quantify the compounds in other biometrics will be more accurate.

In the present study, reverse-phase HPLC with UV diode array detection was developed for the quantification of the intracellular concentration of sesamol. The optimized analytical cell-based assay is relatively easy to do, has few steps, has a quick elution, is highly sensitive, and is precise and accurate. Importantly, the optimized HPLC analysis is cheaper than other analytical tools, such as mass spectroscopy, and is readily available in a wide range of laboratories. Our highly sensitive method provides essential information about a tool to quantify sesamol levels in cell interiors.

## 3. Materials and Methods

### 3.1. Chemicals and Reagents

Sesamol (98% purity) was purchased from Sigma-Aldrich (St. Louis, MO, USA). Methanol (HPLC grade (99.9% purity)) and acetonitrile (HPLC grade (99.9% purity)) were purchased from RCI Labscan (Bangkok, Thailand). Dimethyl sulfoxide (DMSO) (biological grade (purity 99.9%)) was purchased from PenReac AppliChem (Barcelona, Spain). Reagent and culture media, including Dulbecco’s Modified Eagle’s Medium (DMEM) (high glucose), penicillin, streptomycin, and 0.25% trypsin-ethylenediamine tetraacetic acid (EDTA) (1X) were bought from GIBCO^®^, Invitrogen (Grand Island, NY, USA). Fetal bovine serum (FBS) was purchased from GE Life Sciences (Parramatta, Australia). The reagents used in the cell-based assay were of molecular biological grade.

### 3.2. SK-MEL-2 Cell Culture

The human melanoma cell line SK-MEL-2 was purchased from CLS-Cell Lines Service, Eppelheim, Germany. The SK-MEL2 cell line was maintained in DMEM supplemented with 10% fetal bovine serum (FBS) and a solution containing 100 units/mL of penicillin and 100 µg/mL streptomycin. The mixture was incubated at 37 °C in an incubator containing 5% CO_2_. Highly confluent SK-MEL-2 cells in an exponential phase were used in the experiment.

### 3.3. HPLC Stock Solution and Calibration Standards Preparation

A stock solution of sesamol was prepared in methanol and stored at 4 °C until use. The calibration curve for the standard sesamol solution (5, 10, 20, 50, 100, 200, 400, 900, 1000 ng/mL) was done using the stock sesamol solution and HPLC analysis. The standard curve of sesamol was determined each time the experiment was conducted.

### 3.4. Preparation of Quality Control (QC) Sample

SK-MEL-2 cells were cultured in a T-25 flask incubated until full confluency. After incubation, the supernatant was aspirated, and the cells were washed twice with 0.5 mL of 1× ice-cold (4 °C) phosphate buffer solution (pH 7.4). The cells were lysed with 10 mL of methanol and incubated at room temperature for 15 min. Proteins were precipitated out by centrifugation at 13,000× *g* at 4 °C for 15 min. Supernatant (200 µL) was taken and spiked with standard sesamol solutions (20 µL) to get final concentrations of 50, 400, and 900 ng/mL (QC-low, QC-mid, QC-high) prior to HPLC analysis.

### 3.5. Chromatographic Analytical Procedure

The quantitative analysis was performed using a Shimadzu HPLC (LC-2030C-3D, Kyoto, Japan) with a quaternary pump, a column oven, an autosampler, and an UV photodiode array detector (DAD) (Kyoto, Japan). The analysis was performed on a base-deactivated silica (BDS) C-18 reverse-phase column (4.6 × 250 mm, 5-µm packing, Thermo Fisher Scientific, Waltham, MA, USA) with an additional Phenomenex^®^ guard column. The column temperature was set to 30 °C. Methanol and ultrapurified water (70:30, *v*/*v*) filtered through a 0.45-µm Whatman^®^ nylon membrane was used as a mobile phase with a flow rate of 0.8 mL/min. The HPLC injection volume was 20 µL. PDA analyses were performed using a UV spectral wavelength of 297 nm. Labsolution software (version 5.73, Kyoto, Japan) was used for the acquisition of chromatograms, spectra, and integration data.

### 3.6. Method Validation

#### 3.6.1. Selectivity and Carryover Effect

For selectivity, HPLC chromatograms of blank samples (HPLC-grade methanol), cell lysates, 900 ng/mL of the calibration standard, and 900 ng/mL of the QC samples were determined for their respective interference peaks and the sesamol peak. Carryover was investigated by injecting a blank sample after a high concentration of a QC sample (900 ng/mL sesamol).

#### 3.6.2. Recovery

The recovery of sesamol from cell lysates was determined using samples of the QC at three concentrations (QC-low, QC-mid, QC-high) and standard sesamol solutions at similar concentrations (i.e., 50, 400, and 900 ng/mL). The recovery was calculated by comparing the individual sesamol peak areas from the QC samples (*n* = 8 at each concentration) to the average peak areas of the sesamol standard solutions (*n* = 4).

#### 3.6.3. Linearity of the Calibration Curves

Linearity was determined from the calibration curves using the peak area versus the theoretical concentration of the calibration standards (range: 10 to 1000 ng/mL). For each analytical run, the calibration curve was accepted if all of the calibration standards had a deviation within ±15% of the actual concentration, except for with the lowest concentration of the standard curve, the deviation needed to be within ±20% of the actual concentration. A linear regression formula was used to extrapolate the concentrations of sesamol in the QC and in the unknown cell samples after integration of the sesamol peak areas of the corresponding chromatograms.

#### 3.6.4. Limit of Detection and Lowest Limit of Quantification

The lowest concentration of the standard curve was defined as the lowest limit of quantification (LLOQ) of the analysis [22]. The LLOQ was determined from the percent deviation from the experimental concentration, which could not exceed ±20% of the actual concentration: the within- and between-day precision needed to be within ±20% [22]. The limit of detection (LOD) was defined as the lowest concentration where the corresponding peak could be delineated from the background [28]. The LOD was evaluated as the minimum concentration that gave a reliable detection that could be differentiated from the background.

#### 3.6.5. Precision and Accuracy

QC samples were assessed for the precision and accuracy of the analytical method. Each QC sample was prepared and determined in 6 analytical runs on 3 separate days. The percentage of relative standard deviation (% RSD) was calculated to determine the repeatability (within a day) and reproducibility (between days). The deviation of the mean experimental concentration from the nominal concentration, expressed as % bias, was calculated to determine the within- and between-day accuracies. The mean concentration needed to be within ±15% of the actual nominal concentration, which represented the trueness of the analytical method.

### 3.7. Determination of Intracellular Accumulation of Sesamol in SK-MEL-2 Cells

SK-MEL-2 cells were seeded into 24-well plates (2 × 10^5^ cells per well) and incubated at 37 °C in a 5% CO_2_ atmosphere for 24 h. The cell medium was removed, and the cells were pre-incubated with prewarmed (37 °C) Hank’s Balanced Salt Solution (HBSS) pH 7.4 for 20 min. The cells were treated with a sesamol working solution (final concentration of 1 mM) and incubated at 37 °C for 5, 10, 20, 30, and 45 min. At specified intervals, the cells were washed twice with ice-cold (4 °C) PBS and lysed with 200 µL of methanol for 15 min at room temperature. Proteins were precipitated by centrifugation at 2400× *g* at 4 °C for 20 min. The intracellular sesamol concentration was quantified using HPLC, as described above (Section 3.5). The precipitated proteins were resolubilized with 0.1 M sodium hydroxide. The solubilized protein was pipetted for protein determination using a BCA Protein Thermo Fisher Scientific Reagent Kit (Pierce Biotechnology, Rockford, IL, USA). Intracellular unbound sesamol concentrations are presented in nanomoles per milligram (nmol/mg protein).

### 3.8. Correlation between the Intracellular Accumulation of Sesamol and the Antiproliferative Effect in the SK-MEL-2 Cells

Simultaneous antiproliferation and intracellular sesamol concentrations were determined in SK-MEL-2 cells 24 h after treatment. The final concentrations of sesamol were 0.1, 1, 2, 3, and 4 mM. The intracellular accumulation of sesamol was quantified as described in a previous section (Section 3.7).

The antiproliferation of sesamol was performed using a neutral red assay, as previously described, with minor optimization [29]. Briefly, SK-MEL-2 cells were seeded in 96-well plates at 5 × 10^4^ cells per well and incubated for 48 h. Then the cells were treated with sesamol for 24 h. The medium was discarded and incubated with freshly prepared neutral red in medium (final concentration of 50 µg/mL) for 2 h at 37 °C. The supernatant was removed, and then the cells were washed with ice-cold PBS and lysed with 0.33% hydrochloric acid in isopropanol solution. The UV absorbance of neutral red dye was measured by a microplate absorbance reader at 540 nm, and 660 nm was the reference wavelength (Sunrise^TM^, Tecan Austria Gmbh, Grödig, Austria). The results are represented as a percentage of cytotoxicity compared to untreated cells.

### 3.9. Statistical Analysis

Data are represented as mean ± standard deviation. The correlations between the antiproliferative effect and intracellular concentration of sesamol were tested for statistically significant differences through linear regression analysis. All experiments except for method validation were performed in triplicate. The statistical analysis was performed using SPSS 24.0 (SPSS Inc, Chicago, IL, USA), and *p*-values below 0.05 were considered statistically significant. Graphs were plotted using Datagraph 4.3 (Visual Data Tools, Inc, Chapel Hill, NC, USA).

## Figures and Tables

**Table 1:** Deviations from the experimental standard sesamol concentration range (10 to 1000 ng/mL). Data are represented as percent deviations (*n* = 4).

Nominal Conc. (ng/mL)	^a^ Percent Deviations (%)		
	Day 1	Day 2	Day 3
^b^ 10	−4.41	−7.87	−8.78
20	−12.75	−9.10	−6.47
50	5.67	−12.57	2.67
100	−12.69	−14.14	−11.55
200	−8.48	−0.66	−4.28
400	10.44	9.17	9.67
900	0.20	0.34	−5.66
1000	−1.37	−1.54	3.32

^a^ According to FDA recommendations, the deviation should be within ±15% of the actual concentration, while the lowest concentration of the standard curve should be within ±20% of the actual concentration. ^b^ The lowest concentration of the standard curve was found at 10 ng/mL.

**Table 2:** Validation parameters for analytical HPLC method of sesamol, including the retention time (*n* = 10), lower limit of quantification (LLOQ), limit of detection (LOD), and limit of quantification (LOQ).

Validation Parameters	Values
Retention time (min)	4.152 ± 0.008 (0.19% RSD)
LOD (ng/mL)	5
LLOQ (ng/mL)	10 (−8.78% to −4.41% deviation)
Precision of LLOQ (within-days and between-days; %RSD)	3.57% and 7.87% RSD

% RSD: percentage of relative standard deviation. The LOD and LOQ were obtained from four replicates on three separate days.

**Table 3:** Recovery (%) of sesamol in methanol and cell lysate (*n* = 8). QC: quality control.

Concentration (ng/mL)	Recovery (%)
QC-low (50 ng/mL)	94.80 ± 4.69
QC-mid (400 ng/mL)	99.29 ± 3.58
QC-high (900 ng/mL)	99.09 ± 0.84

**Table 4:** Within- and between-day accuracy and precision of HPLC analytical method, expressed as RSD (%) and bias (%) (*n* = 6 and 18 for within-days and between-days, respectively).

Nominal Conc. (ng/mL)	Within-Days				Between-Days					
	Experimental Conc. (ng/mL)	Mean (ng/mL)	RSD (%)	Bias (%)	Experimental Conc. (ng/mL)			Mean (ng/mL)	RSD (%)	Bias (%)
QC-low (50 ng/mL)	46.18	48.32 ± 2.21	4.57	−3.36	52.17	46.18	52.03	49.11 ± 2.05	4.17	−1.78
	48.77				47.15	48.77	49.53			
	46.49				51.81	46.49	50.48			
	51.63				49.61	51.63	48.89			
	50.05				47.27	50.05	46.68			
	46.80				50.39	46.80	48.09			
QC-mid (400 ng/mL)	399.27	392.02 ± 17.44	4.45	−2.00	426.28	399.27	367.33	406.00 ± 21.42	5.28	1.50
	393.54				425.87	393.54	401.74			
	372.18				431.66	372.18	426.13			
	420.87				418.56	420.87	430.98			
	376.70				413.16	376.70	414.39			
	389.54				419.26	389.54	380.61			
QC-high (900 ng/mL)	901.66	896.33 ± 7.50	0.84	−0.41	824.65	901.66	886.03	873.64 ± 34.58	3.96	−2.93
	888.34				825.93	888.34	877.35			
	893.68				832.01	893.68	929.97			
	897.18				831.50	897.18	917.07			
	889.32				835.72	889.32	878.83			
	907.81				828.47	907.81	879.92			

% RSD: Percentage of relative standard deviation.
